# College Satisfaction, Sense of Achievement, Student Happiness and Sense of Belonging of Freshmen in Chinese Private Colleges: Mediation Effect of Emotion Regulation

**DOI:** 10.3390/ijerph182211736

**Published:** 2021-11-09

**Authors:** Jing Tian, Mohan Zhang, Haitao Zhou, Jianfen Wu

**Affiliations:** 1 Zhejiang Academy of Higher Education, Hangzhou Dianzi University, Hangzhou 310018, China; tianjing2018@hdu.edu.cn; 2 Jing Hengyi School of Education, Hangzhou Normal University, Hangzhou 311121, China; zmh@hznu.edu.cn (M.Z.); ppwu70@hznu.edu.cn (J.W.); 3 Faculty of Education, Beijing Normal University, Beijing 100875, China

**Keywords:** sense of belonging, college satisfaction, sense of achievement, emotion regulation, structural equation modeling, Chinese private college

## Abstract

Sense of belonging constitutes a critical component of college students’ retention and academic achievement, especially in disadvantaged higher education institutions such as private colleges in China. Using nationwide survey data (*n* = 3816) from Chinese private colleges, this study explores how college satisfaction, sense of achievement, and student happiness contribute to freshmen’ sense of belonging. Structural equation modeling analyses have identified the significant positive associations between freshmen’s emotion regulation and sense of belonging. In particular, emotion regulation plays a mediating role in the relations between college satisfaction, sense of achievement, student happiness, and the sense of belonging. Therefore, private colleges should design and implement orientation programs to improve freshmen’s learning experience, especially college satisfaction and student happiness, to enhance their sense of belonging.

## 1. Introduction

Private higher education has expanded dramatically worldwide in the last two decades, and China is no exception. Since 2008, the proportion of private colleges in China remained at 28–29% in the higher education sector, and 17.69% of the freshmen in 2019 were enrolled in private colleges [1]. The number of private colleges and their enrolments is rising, with the new student enrollment increase of 7.56%, and the total enrollment of students increase of 11.64% in 2020 [2]. Chinese private colleges have contributed significantly to the development of higher education. However, the rapid expansion of private colleges has caused public concerns and debates about the quality of higher education in China, a country predominantly favoring public universities for decades. Unlike the Ivy League private universities in the United States, Chinese private colleges are usually disadvantaged with low social standing and quality. Accordingly, they lack top talents, financial support, and rights protection; thus, they are generally regarded as the alternatives to public colleges. There are more than 2.44 million postgraduates in China, only 2556 study in private colleges [2]. Moreover, only high school graduates with relatively low tertiary admission scores would consider attending these private colleges. Therefore, the freshmen of these private colleges usually suffer from disadvantages, such as high faculty turnover rate, poor academic performance, educational quality, etc. Consequently, their learning experience and sense of belonging have been substantially compromised, resulting in a high attrition rate in Chinese private colleges. 

This high attrition rate has aroused national concerns about the sustainable development of private colleges in China. Existing studies have found that the facilitating factors and barriers to student retention were closely related to their socioeconomic background, pre-college scoring, social and environmental factors, faculty interaction, academic performance, and student self-efficacy [3,4,5]. Carrol and Birch synthesized those influential factors as situational, attitudinal, and institutional categories [6]. Further studies revealed that attitudinal and institutional categories should be the overwhelmingly advantageous factors to college students’ retention in higher education. The attitudinal factors refer to an individual’s characteristics, such as self-confidence, attitudes, and beliefs [7,8]. More specifically, college students’ sense of belonging to their institution, as an attitudinal factor, could significantly predict their persistence intentions after controlling for background variables and other predictors of persistence [9]. Therefore, enhancing students’ sense of belonging may be a workable strategy to cope with the dropout problems among private college students.

The existing literature on the sense of belonging is focused primarily on students’ demographic characteristics, socioeconomic status, academic engagement, social connections, and life satisfaction. The discourse in the American and European contexts generally involves the working-class, minority, international, and college students with intellectual disabilities, who were all expected to experience more learning difficulties due to lower economic and cultural capital. In contrast, these groups of students attending Chinese private colleges have been neglected and understudied. Understanding how to enhance their sense of belonging will help increase student retention and contribute to the sustainability and competitiveness of private colleges in China. Therefore, this study aims to explore the predictors and mediator(s) of students’ sense of belonging in Chinese private colleges, and in particular, to understand how their emotion regulation mediates their sense of belonging based on personal college experiences (i.e., academic achievement, social life, and student supports). The findings will provide empirical evidence for theoretical development and practical improvement in private colleges in China and other developing countries.

## 2. Conceptual Framework

### 2.1. Antecedents of Sense of Belonging in Learning

According to Strayhorn, a sense of belonging refers to students’ perceived social support on campus, a feeling or a sensation of connectedness, and experiences of mattering or feeling cared about, feeling accepted, respected, valued by, and important to the group or others on campus [10]. Sense of belonging has been identified as a critical outcome of students’ learning experiences in higher education institutions. Previous studies have demonstrated that a sense of belonging positively relates to students’ motivation [11], intention to persist, performance, retention, and graduation [12,13,14,15]. A report from Higher Education Academy concluded that a sense of belonging is at the heart of educational success for all students [16]. Gillen-O’Neel confirmed it as a pivotal factor in study progress, one that substantially impacts students’ everyday lives. “If students experienced a high sense of belonging on a particular day, their emotional and behavioral engagement tended to be higher than usual” [17] (p. 61).

Conversely, a diminished sense of belonging has been associated with risky behaviors, such as emotional distress and even suicidal involvement [18]. Thus, nurturing students’ sense of belonging should positively improve the quality of higher education institutions [19]. Furthermore, students with a strong sense of belonging are likely to experience increased positive psychological functioning and, consequently, increased student performance and college completion rates [20]. Therefore, given the importance of these correlations and their possible impacts, we suggest that colleges need to consider strategies for developing students’ sense of belonging.

Researchers have emphasized the importance of measuring students’ sense of belonging or the extent to which they feel part of the campus community. To that effect, personal status and campus integration were frequently employed [21,22]. Freshmen, or first-year students, are in the transitional period between high school and higher education, and their sense of belonging has been a cynosure for a long time. However, private college students’ experiences during their first year of college are a particularly underexamined area [21]. How they develop their sense of belonging requires further analysis.

Learning experience, including learners’ beliefs and experiences about the learning context, is a clear indicator of students’ academic performance and affects their sense of belonging [23]. Therefore, understanding the key factors affecting students’ sense of belonging, their learning experience, as well as how these factors are linked is necessary to develop students’ positive sense of belonging.

“College satisfaction is a student’s cognitive evaluation of the quality of their college life at a particular institution of higher education” [24] (p. 59); their perceived value, quality, and expectations positively correlate with individual satisfaction [25]. Lekkas et al. suggested that emotion is one of the two determinants of the learning experience, as emotional engagement both positively predicts student satisfaction and moderates the effect of cognitive engagement on satisfaction [26]. Previous studies found that student satisfaction with university services significantly impacts the quality of college life, which subsequently positively influences student loyalty [27]. Student loyalty, almost synonymous with the sense of belonging, helps to retain and graduate students and is critical for the success of both students and higher education institutions. Thus, college satisfaction is an antecedent of a sense of belonging based on the learning experience.

Student happiness is a term that is usually used interchangeably with well-being when researchers investigate students’ subjective positive feelings toward campus learning experience. Moreover, happiness significantly contributes to the sense of community [28]. In higher education institutions, student happiness is another vital factor in facilitating their sense of belonging. Self-esteem, academic success, and financial security explain most of the variance in student happiness [29]. In addition, emotional intelligence, secured attachment patterns, inner-other directedness, and preoccupied attachment patterns contribute significantly to college students’ happiness. For example, Okun et al. found that commitment to college and satisfaction with peer relationships are crucial for first-year college students’ dispositional happiness [24]. Likewise, positive formal teacher–student relationships and friendship experiences promote individual happiness [30]. However, few studies have investigated the relationship between student happiness and the sense of belonging via emotional factors in higher education. This study will fill this gap.

Academic achievement closely relates to college satisfaction and is a psycho-emotional variable of a sense of belonging. The available evidence points to academic achievement as one of the most influential factors of the learning experience. However, apart from the academic, a sense of achievement that illuminates students’ perceived experience and positively correlates with learning variables, such as motivation and deep learning strategies, is in fact anchored student’s learning experience [31]. Although many studies have identified academic achievement as a significant predictor for school satisfaction, student happiness, and sense of belonging in K-12 schools, the impact of the sense of achievement derived from learning experience itself has been understudied, especially in Chinese higher education contexts.

### 2.2. Emotion Regulation

The research focused on college students’ learning experiences has grown significantly through student-centered pedagogy over the past few decades. However, most current analyses of the learning experience are limited to behavioral data and lack other critical dimensions such as learners’ cognitive involvement and emotional experience [32]. Whether the learning experience or the sense of belonging is the psychological term, however, little is known about how learning experiences affect students’ sense of belonging as an internal mental process. Emotion regulation, or how individuals perceive and control their emotions, provides a new perspective to understand this problem, as it relates to cognitive and intelligence factors in the education domain, such as motivation, identity, and well-being [33,34].

As a critical life skill, emotion regulation can facilitate learning and improve educational outcomes [35]. However, research has indicated that university students cannot successfully regulate their emotions during learning, which impedes study progress [36]. The finding that learning experiences provide either positive or negative outcomes depending on the students’ attitude and emotion regulation effectively combines the individuals’ personality traits with the regulatory mechanism that influences learning performance and behavior [26]. Therefore, how emotion regulation facilitates students’ learning experiences needs to be further analyzed. A Chinese study among private college students revealed that emotion regulation is closely related to students’ sense of fulfillment, and emotional adaptation was shown as the most relevant student involvement factor [37]. Additionally, our study aimed to test the role of emotion regulation in private college freshmen’s learning experiences and sense of belonging. Learning experiences included college satisfaction, a sense of achievement, and student happiness.

### 2.3. Conceptual Model and Hypothesis

**Figure 1:**
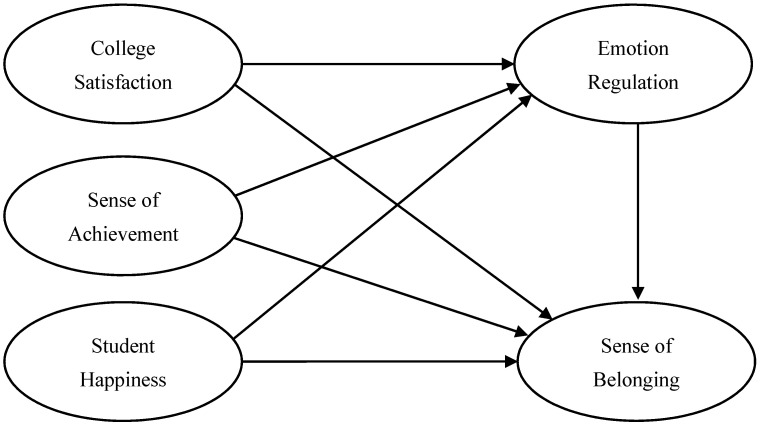
Conceptual model of relationships among college satisfaction, sense of achievement, student happiness, emotion regulation, and sense of belonging.

We developed a conceptual model based on the research-derived relationship among private college freshmen’s college satisfaction, sense of achievement, student happiness and sense of belonging, as illustrated in Figure 1.

Furthermore, we identified two main research questions:

(1) To what extent do college satisfaction, sense of achievement, and student happiness in learning experiences affect private college freshmen’s sense of belonging?

(2) To what extent does emotion regulation mediate the effect of college satisfaction, sense of achievement, and student happiness on private college freshmen’s sense of belonging?

To address those questions, we advanced the following research hypotheses for first-year students in Chinese private colleges:

**Hypothesis** **1a** **(H1a).** *College satisfaction may directly impact private college freshmen’s sense of belonging.*

**Hypothesis** **1b** **(H1b).** *College satisfaction may directly impact emotion.*

**Hypothesis** **1c** **(H1c).** *Emotion regulation mediates the relationship between college satisfaction and a sense of belonging.*

**Hypothesis** **2a** **(H2a).** *Sense of achievement may directly impact private college freshmen’s sense of belonging.*

**Hypothesis** **2b** **(H2b).** *Sense of achievement may directly impact emotion regulation.*

**Hypothesis** **2c** **(H2c).** *Emotion regulation mediates the relationship of sense of achievement and sense of belonging.*

**Hypothesis** **3a** **(H3a).** *Student happiness may directly impact the sense of belonging.*

**Hypothesis** **3b** **(H3b).** *Student happiness may directly impact emotion regulation.*

**Hypothesis** **3c** **(H3c).** *Emotion regulation mediates the relationship between student happiness and a sense of belonging.*

**Hypothesis** **4** **(H4).** *Emotion regulation may directly impact the sense of belonging.*

## 3. Methods

### 3.1. Sample

The data was derived from the 2017 Chinese Private College Students Survey (CPCSS) conducted by the Private Education Research Team of Beijing Normal University. The CPCSS monitored Chinese private college students dynamically to obtain relatively comprehensive information on Chinese private education. A cluster sampling procedure was followed to conduct the survey. The CPCSS project sampled 42,104 students, the population of this study is 3816 freshmen enrolled in the first term in 2017 from certain provinces’ private colleges. Among the total sample, 402 came from municipalities and capital cities of the provinces, 458 came from prefecture-level cities, 990 came from counties, and 1966 came from rural areas; 1337 are the only child, 2479 are non-only children; 2004 have ever acted as the students’ leaders, 1812 have never played the roles of student carders.

### 3.2. Instruments

#### 3.2.1. Emotion Regulation Questionnaire

We modified the Schutte Emotional Intelligence Scale [38] and developed an Emotion Regulation Questionnaire (ERQ) as the instrument. We first collected participants’ demographic information at the beginning of the self-reported ERQ. We then asked for their responses using 20 items designed to measure participants’ ideas on four constructs: an appraisal of emotions, appraisal of others’ emotional empathy, utilization of emotions, and regulation of emotions. The Cronbach’s alpha for the four constructs is 0.812, 0.888, 0.835, 0.879, respectively, indicating satisfactory reliability. The 20 items are measured on a 5-point Likert scale, ranging from “strongly disagree” to “strongly agree”, and the three emotion regulation items adopted from the ERQ were employed in this study.

#### 3.2.2. Learning Experience Questionnaire

The self-reported Learning Experience Questionnaire (LEQ), including 16 items, was employed to understand private college students’ academic and social life experience in colleges. This dependent variable is a measure of subjective experience, which captures students’ evaluation of their learning experience. A 5-point Likert scale was adopted to measure college students’ behavior. Items 1, 2 are sense of belonging, items 3, 4 are sense of involvement, items 5–8 are college satisfaction, items 9–12 are sense of achievement, and items 13–16 are student happiness. The Cronbach’s alpha for the five constructs is 0.805, 0.830, 0.910, 0.885, 0.878, respectively, indicating satisfactory reliability.

#### 3.2.3. Sense of Belonging

College students’ sense of belonging is a multidimensional construct, and it is necessary to distinguish a sense of belonging to peer groups from a sense of belonging at school. Therefore, we adopted the definition from Strayhorn and focused on students’ sense of class belonging and school belonging in Chinese private colleges. Specifically, a sense of belonging was measured by two items (e.g., “You think you are an important part of members of the class”; and “You think the college is a family”). Students’ responses to the questions were scaled on a 5-point Likert scale. As shown in Table 1, its Cronbach’s alpha is 0.807, indicating satisfactory reliability.

#### 3.2.4. College Satisfaction

Bowman and Culver computed college satisfaction using college students’ evaluation of their entire educational experience and willingness to recommend the institution [39]. The College Satisfaction Scale measured the degree of college satisfaction in the study, choice, utility for career, relationships, and service [40]. Following standard practices for sampling for college satisfaction, four general statements were generated, including climate, academic, administration, and service. Students rated their conformity level on the 5-point Likert scale, ranging from “strongly disagree” to “strongly agree”. The four items are as follows: “You think your college loves students”; “You think your program’s cultivating plan is appropriate”; “You think the management of your college is reasonable”; and “You think the student service is high-quality and effective of your college”. As shown in Table 1, its Cronbach’s alpha is 0.911, indicating satisfactory reliability.

#### 3.2.5. Sense of Achievement

Academic achievement is usually measured using a cumulative grade point average. Sense of achievement describes a subjective perception of academic achievement. This study focuses on college students’ perception of achievement. Sense of achievement was measured using two items: “I learn a lot”; “I am excited because I’ve learned professional knowledge”. These two items were examined on a 5-point Likert scale. As shown in Table 1, its Cronbach’s alpha is 0.825, indicating satisfactory reliability.

#### 3.2.6. Student Happiness

Happiness as perceived positive emotions was measured through questions designed to rate happiness. Seligman’s PERMA model consists of five elements of happiness: positive emotions, engagement, relationships, meaning, and accomplishment [41]. Previous studies employed emotions such as confidence, interest, and excitement to measure psychological experience in students’ learning experience. Therefore, we used three items to measure student happiness, they are as follows: “You think your college life is very happy”; “You are satisfied when you have a good friendship with your classmate”; and “You are gratified when you do something meaningful at college”. These items were examined on a 5-point Likert scale, ranging from “strongly disagree” to “strongly agree”. As shown in Table 1, its Cronbach’s alpha is 0.851, indicating satisfactory reliability.

### 3.3. Data Analysis

With the SmartPLS software, we developed the Partial Least Squares Structural Equation Modelling (PLS-SEM) for the empirical analysis to examine the hypotheses. PLS-SEM computes determinate latent variable scores [42]. The advantages of SEM using SmartPLS are: (a) creates out-of-sample predictions, (b) utilizes composites scores in additional analyses, (c) offers great flexibility, (d) does not suffer from factor indeterminacy, and (e) variance-based SEM yields robust results [43]. In addition, PLS can be used for theory confirmation, suggesting where relationships might or might not exist and suggesting propositions for testing later [44]. SmartPLS 3 combines state-of-the-art methods with an easy-to-use and intuitive graphical user interface [45]. Previous studies employed SmartPLS software to examine servant leadership, career, and life satisfaction [46], student satisfaction, and loyalty in higher education [47]. Likewise, we analysed the database using SmartPLS 3.3 software (SmartPLS GmbH, Boenningstedt, Germany).

We tested the normality of the five constructs: college satisfaction, sense of achievement, student happiness, emotion regulation, and sense of belonging. Skewness and kurtosis coefficients could provide a powerful scheme for assessing normality against a wide variety of alternative distributions [48]. The current study’s skewness and kurtosis values range from −0.516 to −0.266 and from 0.423 to 1.222, respectively, and the values are in the ranges between |3| and |8|.

Casual Step Approach was conducted to examine model hypotheses. According to this approach, we should fit three regressions equations:

Y = cX + e1(1)

M = aX + e2(2)

Y = c’X + bM + e3(3)

Subsequently, (i) Examining the coefficient c of Equation (1), that is, examining H0: c = 0. (ii) Examining the coefficient a of Equation (2), that is, examining H0: a = 0. (iii) Examining the coefficient b of Equation (3), that is, examining H0: b = 0. If coefficient c of (i) is significant, coefficient a and b of (ii) and (iii) are significant, and we can conclude that the medicating effect is significant. The full mediation process should add the coefficient c’ of (iii) and Equation (3) is not significant. After having approaches for coefficients a and b, the coefficient c, which was initially significant, turns insignificant c’ [49].

## 4. Results

### 4.1. Measurement Model Assessment

The Cronbach’s alpha, average variance extracted (AVE), composite reliability (CR), and discriminant validity are illustrated in Table 1.

As illustrated in Table 1, all items from the learning experience questionnaire had factor loading values over 0.8, which are greater than 0.4, so all the items are reserved [50]. We used the average variance extracted (AVE) to evaluate the convergent validity; AVE equal to or above 0.5 shows high convergent validity [51]. The composite reliability (CR) above 0.7 indicated a high internal consistency [52]. Table 1 illustrates that all AVE construct values are over 0.7 and all CR construct values are over 0.9. Therefore, we retained those items in the learning experience.

Table 2 shows the instrument’s discriminant validity. The square root of AVE for each of the constructs is more than the correlations’ estimate of the constructs represents a good discriminant validity. As outlined in Table 2, all constructs’ discriminant validity meets this rule, indicating a good discriminant validity for the constructs.

The standardized root mean square residual (SRMR) and normed fit index (NFI) were used to test the model fit in SmartPLS3.3. When the SRMR value is smaller than 0.11, and the NFI is above 0.8 means, the model fits [53]. For example, the SRMR value is 0.069, and the NFI value is 0.815 for this model, we can conclude that the model has a satisfactory fit.

### 4.2. Structural Model Assessment

This model’s path coefficients were estimated using the bootstrapping technique with 1000 resamples, as illustrated in Table 3. A structural model was conducted to estimate structural path coefficients among college satisfaction, sense of achievement, student happiness as independent variables, and sense of belonging as dependent variables. As presented in Table 3, all the hypothesis paths were significantly supported except Hypothesis H2a. The structural model assessment revealed that freshmen’ sense of belonging is significantly impacted by college satisfaction (*β* = 0.379), student happiness (*β* = 0.262), and emotion regulation (*β* = 0.209). The results also revealed that first-year students’ emotion regulation is significantly influenced by college satisfaction (*β* = 0.120), sense of achievement (*β* = 0.209), and student happiness (*β* = 0.312).

To examine the proposed mediation model, a two-step bootstrapping procedure was used. First, we assessed the total effects of college satisfaction, sense of achievement, student happiness, and sense of belonging on a sense of belonging without mediator variables. College satisfaction (*β* = 0.405), sense of achievement (*β* = 0.092), and student happiness (*β* = 0.327) was significantly related with Sense of Belonging (Table 4).

**Figure 2:**
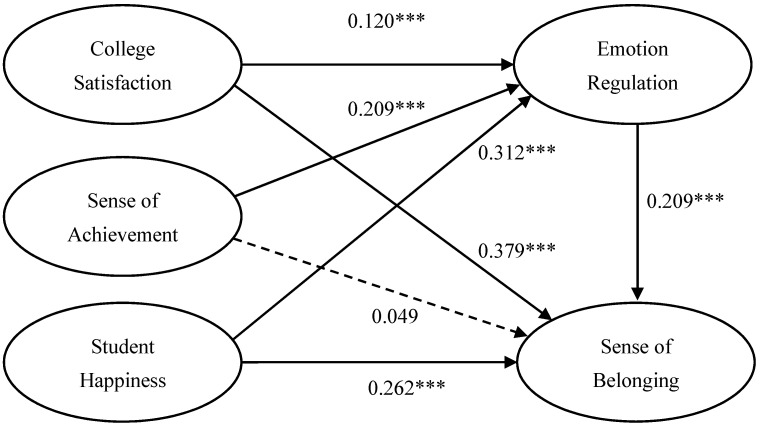
Structural model of relationships among College Satisfaction, Sense of Achievement, Student Happiness, emotion regulation, and sense of belonging. Dashed line signifies non-significant paths (*p* > 0.05). *** *p*< 0.001.

Second, we added emotion regulation as the mediator variable, and the three path coefficients of college satisfaction, sense of achievement, and student happiness declined. As illustrated in Table 3 and Table 4, the relationship between college satisfaction and sense of belonging is still significant, and the path coefficient declined from 0.405 to 0.379. Likewise, the relationship between student happiness and sense of belonging is still significant, and the path coefficient declined from 0.327 to 0.262. Nevertheless, the relationship between sense of achievement and sense of belonging turns insignificant, and the path coefficient declines from 0.092 to 0.049. Thus, with emotion regulation as a mediator, the indirect effects between college satisfaction, sense of achievement, student happiness, and sense of belonging are supported. Accordingly, the full mediation of emotion regulation on the relationship between sense of achievement and sense of belonging was supported, as illustrated in Figure 2.

## 5. Discussion

This study aimed to investigate the effects of learning experiences, including college satisfaction, sense of achievement, and student happiness, on the sense of belonging. We also tested the extent to which emotion regulation mediates college satisfaction, sense of achievement, student happiness, and the sense of belonging.

### 5.1. The Effect of College Satisfaction

The finding demonstrates that college satisfaction positively affects freshmen’ sense of belonging in Chinese private colleges. This study suggests that students with a greater sense of satisfaction with college may have a greater sense of belonging [54], consistent with other higher education findings conducted in Western countries. We also assessed how college satisfaction affects the sense of belonging inversely. College satisfaction positively affects emotion regulation for first-year students in Chinese private colleges, similar to previous studies’ findings. For example, satisfaction with university services correlates with nonintellectual competencies, such as peer relationships, mood monitoring, self-esteem, and external motivation.

When emotion regulation is a mediator, the impact of college satisfaction on a sense of belonging remains significant, but with a decrease in the path coefficient, which means emotion regulation is the mediating variable of college satisfaction and sense of belonging. Thus, emotion regulation partially mediates the effect of college satisfaction on the sense of belonging. Therefore, college satisfaction positively affects students’ sense of belonging, while emotion regulation plays a partial mediation role.

### 5.2. The Effect of Sense of Achievement

As supported by the structural model used in this study, there was a significant total effect of students’ sense of achievement on their sense of belonging for Chinese private college freshmen. This echoes the prevalent conclusion that student academic achievement significantly relates to the sense of belonging [55]. This study emphasized students’ satisfaction perception based on the academic experience at college, and the results are consistent with findings on GPA [56]. Not all academic achievements can be assessed by the existing performance evaluation system, for instance, individual, cognitive development, practical ability, and social competence. This study focuses on students’ perceived achievement and contributes to the literature by finding a positive relationship between a sense of achievement and a sense of belonging.

Our study’s key finding is that emotion regulation fully mediated the effects of achievement on the sense of belonging for first-year students at Chinese private colleges. Before adding emotion regulation, sense of achievement significantly impacts freshmen’ sense of belonging; however, the path coefficient declines significantly after adding emotion regulation as the mediator. Simultaneously, the direct effect between a sense of achievement and belonging changes from significant to insignificant. This finding replicates previous research showing that academic achievement is based on a combination of cognitive ability and personality traits [57]. The finding also demonstrates that a sense of achievement may positively affect a sense of belonging by influencing college students’ emotion regulation.

### 5.3. The Effect of Student Happiness

Although many studies have focused on students’ happiness in higher education, little attention has been given to the relationship between students’ happiness and their sense of belonging. This study demonstrates that college students’ happiness has a direct impact on their sense of belonging. Furthermore, this study revealed the partial mediation between student happiness and sense of belonging by regarding emotion regulation as a mediator. Research has indicated that, in primary school, the sense of belonging is a strong indicator of children’s happiness [58]. Our study extended this finding by examining the impact between student happiness and sense of belonging in the context of higher education. Additionally, this study contributes to the literature by highlighting the significant effect of student happiness on their sense of belonging through emotion regulation as a mediator.

### 5.4. Emotion Regulation and Sense of Belonging

Based on Luo et al.’s finding that Chinese students had high levels of emotional engagement [59], this study revealed the relationship between students’ emotion regulation and sense of belonging in Chinese private colleges. Both academic and non-academic factors influence students’ sense of belonging [44]. For example, using emotional teaching strategies to develop students’ sense of belonging can foster learning [60]. Combined with this study’s findings, developing college students’ emotion regulation level directly relates to their sense of belonging. Interestingly, this study finds that emotion regulation plays an important mediation role between the learning experience and sense of belonging as a cognitive factor.

## 6. Conclusions

This study provides a quantitative insight into the issues of freshmen’ sense of belonging at Chinese private colleges. It explores how first-year students belong to their college communities. The result demonstrates that college satisfaction, sense of achievement, and student happiness affect the sense of belonging. This result aligns with the previous study, which has identified the positive relationship between the learning environment and a sense of belonging. This study emphasizes the importance of improving college students’ learning experience in developing a sense of belonging. Freshmen’s sense of belonging would be enhanced when instruction is well designed and implemented. This requires active management of the student-college relationship to promote students’ sense of belonging through improving college satisfaction and student happiness. Considering the importance of emotion regulation in mediating the sense of belonging in Chinese private colleges, students’ positive emotion regulation needs to be developed to reinforce their sense of belonging. As emotion regulation is often a challenge for college students, the strategy requires careful consideration of students’ academic and non-academic perceptions. By understanding the connecting roles of emotion regulation and a sense of belonging, dynamic and functional campus environments can be developed. College management must address and support students’ satisfaction, student relations on campus, and academic and non-academic activities to enhance students’ happiness and sense of achievement to improve their sense of belonging.

## 7. Limitations and Future Research

The results of the present study need to be noted in light of several limitations. First, self-reported measures for learning experience and sense of belonging create a social desirability bias risk. As qualitative research is both exploratory and descriptive and provides intersections of personal narratives to develop meaning, a mixed-method approach is encouraged to be employed to decrease the risk of socially desirable answers. Second, this study’s findings were limited to Chinese private college students and cannot be generalized to K-12 or Chinese public colleges. Further studies among other institutions are necessary to validate and extend these findings. The study could be undertaken internationally to develop more large-scale results.

## Figures and Tables

**Table 1:** Summary of the measurement model.

Latent Variables	Items	Outer Loading	AVE	CR	Cronbach’s Alpha
College Satisfaction	LEQ5	0.885	0.788	0.937	0.911
	LEQ6	0.867			
	LEQ7	0.893			
	LEQ8	0.907			
Sense of Achievement	LEQ9	0.928	0.851	0.920	0.825
	LEQ10	0.918			
Student Happiness	LEQ13	0.840	0.771	0.910	0.851
	LEQ15	0.903			
	LEQ16	0.890			
Emotion Regulation	ERQ18	0.881	0.806	0.926	0.879
	ERQ19	0.905			
	ERQ20	0.906			
Sense of Belonging	LEQ1	0.909	0.838	0.912	0.807
	LEQ2	0.922			

Note: AVE = Average Variance Extracted, CR = Composite Reliability.

**Table 2:** The square root of AVE and bivariate correlations between the constructs.

	CS	SA	SH	ER	SB
College Satisfaction (CS)	**0.888**				
Sense of Achievement (SA)	0.726	**0.923**			
Student Happiness (SH)	0.605	0.709	**0.709**		
Emotion Regulation (ER)	0.460	0.517	0.532	**0.898**	
Sense of Belonging (SB)	0.669	0.618	0.637	0.548	**0.910**

Note: Diagonal elements highlighted in bold represent the square root of AVE. Off diagonal elements are bivariate correlations between the constructs.

**Table 3:** Structural model assessment.

Hypothesis Pathway	Path Coefficient	T-Value	Result
H1a: college satisfaction → sense of belonging	0.379	18.081 ***	Supported
H1b: college satisfaction → emotion regulation	0.120	5.265 ***	Supported
H2a: sense of achievement → sense of belonging	0.049	1.907	Not supported
H2b: sense of achievement → emotion regulation	0.209	7.085 ***	Supported
H3a: student happiness → sense of belonging	0.262	12.513 ***	Supported
H3b: student happiness → emotion regulation	0.312	12.733 ***	Supported
H4: emotion regulation → sense of belonging	0.209	12.114 ***	Supported

Note: *** *p* < 0.001.

**Table 4:** Mediation effect results.

Hypothesis Pathway	Path Coefficient	T-Value	Result
**Total Effects**			
college satisfaction → sense of belonging	0.405	18.564 ***	-
sense of achievement → sense of belonging	0.092	3.426 **	-
student happiness → sense of belonging	0.327	15.324 ***	-
**Indirect effects**			
H1c: college satisfaction → emotion regulation → sense of belonging	0.025	4.847 ***	Supported
H2c: sense of achievement → emotion regulation → sense of belonging	0.044	5.871 ***	Supported
H3c: student happiness → emotion regulation → sense of belonging	0.065	8.982 ***	Supported

Note: *** *p* < 0.001. ** *p* < 0.01.

## Data Availability

According to the data access policies, the data used to support the findings of this study are available from Beijing Normal University, upon a reasonable request made by email: zht@bnu.edu.cn.
